# Structural measurement of electron-phonon coupling and electronic thermal transport across a metal-semiconductor interface

**DOI:** 10.1038/s41598-022-20715-5

**Published:** 2022-10-05

**Authors:** Wonhyuk Jo, Jungyun Kee, Kooktea Kim, Eric C. Landahl, Grace Longbons, Donald A. Walko, Haidan Wen, Dong Ryeol Lee, Sooheyong Lee

**Affiliations:** 1 Korea Research Institute of Standards and Science, Daejeon, 305-340 South Korea; 2 Department of Physics, Soongsil University, Seoul, 06978 South Korea; 3 Center for Spintronics, Korea Institute of Science and Technology, Seoul, 02792 South Korea; 4 Department of Physics and Astrophysics, DePaul University, Chicago, IL 60614 USA; 5 Advanced Photon Source, Argonne National Laboratory, Lemont, IL 60439 USA; 6 Materials Science Division, Argonne National Laboratory, Lemont, IL 60439 USA; 7 Department of Nanoscience, University of Science and Technology, Daejeon, 305-340 South Korea; 8 Present Address: European X-ray Free Electron Laser GmbH, Schenefeld, 22869 Germany; 9 Present Address: Physics Department, University of Notre Dame, Notre Dame, IN USA

**Keywords:** Nanoscale materials, Applied physics, Condensed-matter physics, Electronics, photonics and device physics, Techniques and instrumentation

## Abstract

Scattering of energetic charge carriers and their coupling to lattice vibrations (phonons) in dielectric materials and semiconductors are crucial processes that determine the functional limits of optoelectronics, photovoltaics, and photocatalysts. The strength of these energy exchanges is often described by the electron-phonon coupling coefficient, which is difficult to measure due to the microscopic time- and length-scales involved. In the present study, we propose an alternate means to quantify the coupling parameter along with thermal boundary resistance and electron conductivity by performing a high angular-resolution time-resolved X-ray diffraction measurement of propagating lattice deformation following laser excitation of a nanoscale, polycrystalline metal film on a semiconductor substrate. Our data present direct experimental evidence for identifying the ballistic and diffusive transport components occurring at the interface, where only the latter participates in thermal diffusion. This approach provides a robust measurement that can be applied to investigate microscopic energy transport in various solid-state materials.

## Introduction

Light-matter interactions involving energetic electrons and quasiparticles are of fundamental interest in condensed matter physics^1,2^ and are also critical to the development of next-generation microelectronics. In particular, the interaction between electronic states and the lattice in excited solids stands out as one of the dominant scattering processes that determine various material properties, ranging from charge carrier and thermal transport, high-temperature superconductivity^3,4^, and charge density waves^5^. Despite such importance, characterization of electron-phonon coupling (EPC) is challenging, especially when the system is driven out of thermodynamic equilibrium. The essence of EPC lies in the fact that perturbation of the electronic state leads to an exchange of energy and momentum between electrons and phonons, and therefore results in elastic stress. Ultrafast optical excitation can induce such transient states, in which a large population of hot carriers is generated and relaxes its excess energy to the cold lattice. Therefore, atomic-scale measurements of structural changes in relevant timescales potentially allow us to explore fine details of the coupling between the electronic states and the lattice.

The capability to deliver femtosecond laser pulses on metallic surfaces provides a systematic means to induce strong non-equilibrium between electron and phonon subsystems. Typically, the EPC coefficients can be measured by using time-domain thermo-reflectance methods^6–9^, where the sample’s surface reflectively is monitored after the incidence of femtosecond laser pulses. The time-dependent reflectance is compared with the two-step thermal relaxation model^10^ that assumes the linear relation between the reflectivity and temperature. However, the experimentally acquired values have often shown considerable variation likely due to the complex interplay between electrons and phonons as well as multiple scattering mechanisms. To mitigate such experimental challenges, first principle calculations have been conducted on metals and semiconductors to confirm the EPC coefficients^11^. Nevertheless, the availability of such results is still limited and their ability to account for discrepancies between the simulation and the experiments remains questionable^12^.

In this work, we conduct high angular resolution and large dynamic range time-resolved X-ray diffraction (TRXD) measurements of transient lattice displacement across buried layers of optically excited metal-to-semiconductor heterostructures, such as Cr/GaAs and Cr/Si. Obtaining the laser-induced changes in diffraction patterns from the substrates over a wide *Q*-range is especially important for modeling femtometer-scale lattice deformation in solids. The long material penetration depth of hard X-rays also enables probing propagation of impulsively driven strains from the thin metal surface to the bulk semiconductor, which contains information about athermal (ballistic) contribution of phonons. In our X-ray measurement, the ballistic components appear in the form of transient interference fringes in reciprocal space or X-ray Brillouin scattering. In order to quantify the relevant physical parameters (e.g., EPC coefficient, thermal conductivity of electrons and lattice), we perform a time- and depth-dependent strain analysis by using dynamical X-ray diffraction theory^13–15^ and the heat transport model. By comparing the experimental result to the modeling, we determine the EPC coefficient *G* of 84 $\times$ $10^{16}$ Wm$^{-3}$K$^{-1}$ for the Chromium metal layer, which is twice as large as previously reported^16,17^. Furthermore, we find an effective thermal conductivity of the electrons $k_e$ of 18.7 W m$^{-1}$K$^{-1}$, which is nearly 5 times smaller than the bulk value^18^. We attribute these apparent discrepancies to the effects of high electron temperatures following intense laser irradiation as well as multiple electron scattering events taking place at the grain boundaries in the metal film. Our structural measurement yields thermal conductivities of the substrates $k_s$ of 34 and 5.1 Wm$^{-1}$K$^{-1}$ for the Silicon (Si) and Gallium Arsenide (GaAs), respectively. These values are nearly an order of magnitude smaller than the bulk thermal conductivities of the respective materials. Such difference may be caused by the predominant roles of the quasi-ballistic thermal transport mechanism near the interface.

**Figure 1:**
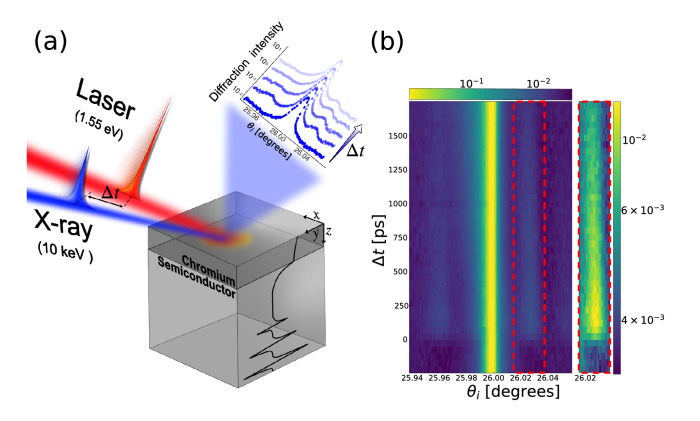
(**a**) Schematic of the TRXD experiment of metal layered semiconductor, and (**b**) time- and angle-resolved intensity profiles of the obtained GaAs (0 0 4) Bragg diffraction peak. The red dashed box indicates the region of interest where the intensity modulation along the angular axis is detected due to X-ray Brillouin scattering.

## Results and discussion

### Structural dynamics upon optical excitation

Temporal evolution of X-ray diffraction curves from the symmetric (0 0 4) Bragg reflection of a laser-excited GaAs substrate is shown in Fig. 1b. We note that the metal film absorbs most of the optical photons from the pump laser beam within its absorption length (approximately 18.5 nm at 800 nm), which is significantly shorter than the film thickness of 80 nm. Therefore, the transient distortions of the Bragg peak of the semiconductors are entirely caused by the strains that migrate from the metallic film. The optically induced strain in the substrate is comprised of two sources of elastic distortion. First, rapid lattice response at relatively early time-scales (< 1 ns) is caused by propagating longitudinal sound waves (coherent phonons) from the film. More specifically, the initial expansion of the lattice in the film layer is followed by a compression effect as the impulsive bipolar strain propagates away from the surface at the speed of sound. Due to the acoustic impedance mismatch at the interface, the sound wave splits into transmitted and reflected components, with contributions that can be described by an acoustic mismatch model^19^. The reflected acoustic wave makes multiple round trips between the film surface and the interface while its amplitude continuously decreases^20,21^. Consequently, we observe the emergence of a phonon-mediated real-space periodicity in the substrate crystal as evidenced by the strong sidebands at $\Delta \theta$ = 61 and 98 arcseconds from Si and GaAs, respectively, near the main crystal Bragg diffraction peak. Second, we expect an additional lattice distortion due to thermal diffusion from the film layer into the substrate. At time delays greater than 1 ns, we observe a slight intensity increase in the lower angle side of the Bragg peak resulting in an asymmetric shape of the curve. The inhomogeneous temperature distribution $\Delta$T(z) within the buried layer is the likely cause of this deformation of the X-ray diffraction peak. In order to extract more quantitative information about the strain evolution, we modeled the depth- and time-dependent strain profiles of the substrate by solving the numerical partial differential equations (PDE) as shown in the following section.

### Two temperature model and impulsive pulse train

The phenomenological temperature relationship between the electrons $T_e$ and the lattice $T_l$ is described by the two temperature model^22^ as follows:

1a $$\begin{aligned} C_{e} \frac{\partial T_e}{\partial t}&= k_e\frac{\partial ^2 T_e}{\partial z^2} - G(T_e - T_l) + S(z,t=0) \end{aligned}$$

1b $$\begin{aligned} C_{l} \frac{\partial T_l}{\partial t}&= k_l\frac{\partial ^2 T_l}{\partial z^2} + G(T_e - T_l), \end{aligned}$$

where $k_e$ and $k_l$ are thermal conductivities and $C_e$ and $C_l$ denote volumetric heat capacities of the electrons and the lattice, respectively. The strain profile reduces to a one-dimensional case over the thickness of the X-ray penetration depth into the material because the measurement is primarily sensitive to the changes along the surface normal direction in our experimental geometry. The source term *S* is given as $\frac{F(1-R)}{C_e \delta } e^{-z/\delta }$, where *F*, *R*, and $\delta$ denote fluence, reflectance, and optical penetration depth of the pump laser in the film layer, respectively. In our work, we employed the values of *F* = 8 mJ/cm$^2$, *R* = 0.6, and $\delta$ = 18 nm. We note that the higher pump fluence would lead to faster thermalization due to higher energy electrons scattering more often according to kinetic theory. Consequently, the heat energy would be more localized at the surface of the metal thin film. This initial temperature profile will initiate a narrower and stronger impulsive pulse train propagating into the substrate. The first term in the right-hand side of Eq. (1b) is normally negligible since $k_l<< k_e$ in metals^17,23^. Here, we solve the partial differential equations numerically by using finite difference methods^24^. We assume that the electronic heat capacity carries a linear relationship with the electron temperature $C_e(T_e) = \gamma T_e$, where $\gamma$ is a constant value when the thermal energy of electrons at $T_e$ is smaller than the Fermi energy (6.9 eV for Cr)^6^. Essentially, this equation describes the equilibration process, in which the electron subsystem continuously imparts its excess energy to the lattice until reaching the same temperature. Simultaneously, the electrons and phonons diffuse into a cold region.

**Figure 2:**
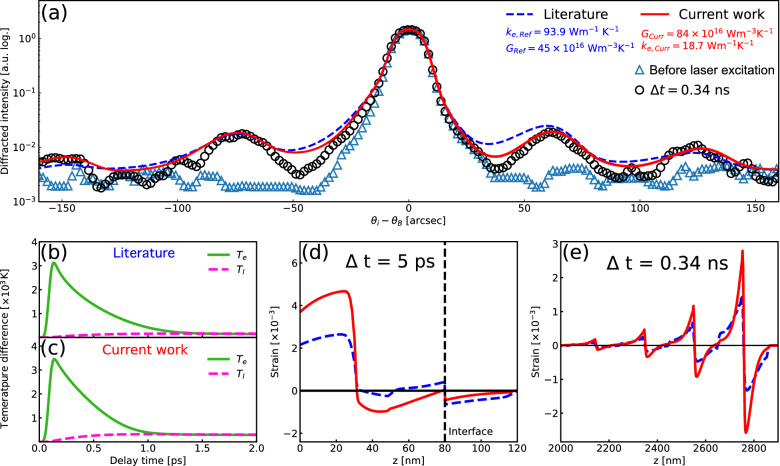
(**a**) Si (004) rocking curve (black circle) and calculation result of dynamical diffraction theory (blue dashed and red solid lines based on the literature values for *G* and $k_{e}$ and current work). The two temperature model for electron temperature ($T_e$) and lattice temperature ($T_l$) following laser excitation are shown for the literature values (**b**) and the current work (**c**). The calculated strain profiles for both the literature values (dashed blue) and current work (red solid) were solved at $\Delta$t = 5 ps (**d**) and at $\Delta$t = 0.34 ps (**e**).

In our strain model, the impulsively generated acoustic phonon propagates into the depth direction with a speed of sound in Cr ($v_{Cr}$ = 6608 m/s), and the strain pulse is partially reflected at the interface due to the impedance mismatch. Since the air-metal interface also acts as another reflector for the back-propagating pulse, the single impulsive pulse induces a train of pulses in the substrate material (see Fig. 2e)^20,21^. The effect of this strain profile is manifested in the diffracted X-ray intensity as a fringe pattern near the vicinity of the main Bragg peak, which is a characteristic feature of Brillouin scattering^25,26^. Figure 2a shows the Si (0 0 4) rocking curve and the fitting result based on the dynamical X-ray diffraction theory as a function of angles that are shifted based on the Bragg condition at equilibrium ($\theta _{\text {B}} = sin^{-1}(\frac{n \lambda }{2d})$). According to the Brillouin scattering condition^25,26^, the fringe spacing in reciprocal space corresponds to the periodicity of the impulsive pulse train in real space in Fig. 2e and thus is proportional to the film thickness. We confirm that this thickness analysis result is consistent with the X-ray reflectivity measurements within 10 $\%$ (see Fig. 4c).

Figure 2a compares the high-resolution X-ray diffraction curve from Si (0 0 4) reflection measured at a time-delay of $\Delta$t = 0.34 ns to the modeling results (red solid line), which are constructed by adjusting the *G* and $k_e$ values while simultaneously matching the absorbed laser fluence found from the average lattice thermal expansion. Fitting the rocking curve shape using these two adjustable parameters shows better agreement with the experimental data as compared to the model based on the previously reported values (blue dashed line)^16,17^, which does not reproduce the clear Brillouin scattering fringe pattern in both its location and amplitude. The fine details of the Brillouin scattering fringes are sensitive to the shape and amplitude of the acoustic wave propagating across the material’s interface, of which features depend on its generation mechanisms. To gain additional insight into how the shape and amplitude of the acoustic waves affect the Brillouin scattering fringe pattern, we calculate the initial electron and lattice thermalization profiles based on *G* and $k_e$ values from previous reports^27^ as well as our current work. As shown in Fig. 2b and c, our result yields a thermalization time of 1.2 ps that is slightly less than the reference values of 1.5 ps. Here, the thermalization times are estimated from the time points at which the temperature difference ($T_e - T_l$) approaches zero. A faster energy equilibriation between electron and phonon subsystems in our case leads to more pronounced strain responses in the film as shown in Fig. 2d at $\Delta t$ = 5 ps. It also generates a pulse train with higher peak-to-peak amplitudes in the substrate as shown in Fig 2e at $\Delta t$ = 0.34 ns. Consquently, we find that a faster lattice thermalization in the metalic film is necessary to correctly reproduce the fine details of the Brillouin scattering patterns in the vicinity of the Bragg peaks. Comparing our modeling result to the experimental data for the time delay of $\Delta t$ = 340 ps yields the *G* and $k_e$ values of 84 $\times 10^{16}$ Wm$^{-3}$K$^{-1}$ and 0.94 W m$^{-1}$K$^{-1}$, respectively. A calculated estimate for the EPC of Cr based on the free electron theory yields approximately 45 $\times 10^{16}$ Wm$^{-3}$K$^{-1}$^17^. Previous optical reflectivity measurements also reported a similar result of *G* = 42 ± 5 $\times 10^{16}$ Wm$^{-3}$K$^{-1}$^16^.

## Discussion

### Thermal boundary conductance and conductivity

The effect of phonon transport and electron-phonon coupling that determine the heat transport at the solid-solid interface has been a long standing problem in condensed matter science both experimentally and theoretically^28^ In order to understand the thermal boundary conductance playing an important role in heat transport between materials, we employ the Fourier’s heat transfer model to describe the thermal transport process across the metal-to-semiconductor interface where the metallic film is initially excited by a femtosecond laser pulse and heated via lattice thermalization^29^. In this case, the thermal diffusion equation can be given as

2a $$\begin{aligned} C_f\frac{\partial T_f}{\partial t}&= k_f\frac{\partial ^2 T_f}{\partial z^2}, \end{aligned}$$

2b $$\begin{aligned} C_s\frac{\partial T_s}{\partial t}&= k_s\frac{\partial ^2 T_s}{\partial z^2}, \end{aligned}$$

where $C_f$ and $C_s$ are volumetric heat capacities of film and substrate, and $k_f$ and $k_s$ are thermal conductivities of film and substrate, respectively. In our case, $k_f$ corresponds to $k_l$ in Eq. (1). We consider a one-dimensional diffusion because the pump laser beam size is at least 40 times larger than the X-ray probe on the sample surface where the thermal gradient along the depth direction should be significantly greater than along the lateral direction. The initial temperature distribution in the substrate is given as $T_s(z) = 0$. Here, we extract the thermal boundary conductance $\sigma _{TBC}$ by solving Eq. (2) with the following boundary conditions:

3a $$\begin{aligned} -k_f\frac{\partial T_f}{\partial z}\bigg |_{z=d}&= \sigma _{TBC}(T_f - T_p), \end{aligned}$$

3b $$\begin{aligned} -k_s\frac{\partial T_s}{\partial z}\bigg |_{z=d}&= \sigma _{TBC}(T_f - T_p), \end{aligned}$$

3c $$\begin{aligned} \frac{\partial T_f}{\partial z}\bigg |_{z=0}&= 0. \end{aligned}$$

**Figure 3:**
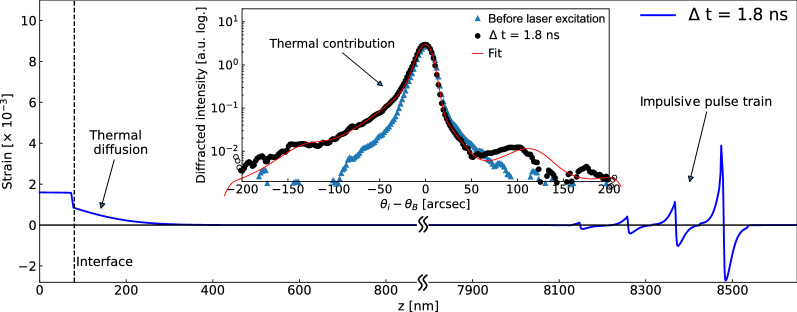
Strain profile of GaAs substrate at $\Delta t = 1.8$ ns, and corresponding Bragg diffraction curve together with a fit result (inset). The non-homogeneous temperature distribution near the interface gives rise to the asymmetry of the curve to the part of the lower angle.

The finite difference method^24^ is used to solve the PDE numerically to estimate the temperature distribution as a function of time. Subsequently, the temperature distribution in the bulk substrate is converted to the strain profile by using a linear thermal expansion coefficient $\alpha$. We note that the transient strain generated in the substrate layer at early time-scales pertains necessary information to extract $k_f$, $k_s$ and $\sigma _{TBC}$ values. As can be seen in Fig. 3, a clear asymmetry in the Bragg diffraction peak from the semiconductor layer reflects the presence of the inhomogeneous strain profile arising from the thermal gradient near the interface. By comparing the numerical model to data, we obtain $\sigma _{TBC}$ and $k_s$ values for the Cr layered Si and GaAs semiconductors as shown in Table 1.

**Table 1:** Fitting parameters of thermal boundary conductivity and thermal conductivity of the samples.

Substrate	Si		GaAs	
Cr thickness [nm]	80		80	
Method	Current work	Ref.	Current work	Ref.
$\sigma _{TBC}$ ($\times 10^8$ Wm$^{-2}$K$^{-1}$)	1.1	2.0^29^	0.5	–
$k_s$ (Wm$^{-1}$K$^{-1}$)	34 $\pm 4.27 \%$	148^29^	5.1 $\pm 0.86 \%$	55^30^

### Electron-phonon coupling coefficient

The electron-phonon coupling factor *G* in Eq. (1) has been widely used to model ultrafast laser heating processes^31^ and is given as follows:

4 $$\begin{aligned} G = \frac{\pi ^2}{6}\frac{m_e V_s^2 n_0}{\tau _e(T_e) T_e}, \end{aligned}$$

where $m_e$, $V_s$, and $n_0$ are the effective mass of electron, speed of sound, and number density of electrons, respectively. Anisimov et al.^22^ has assumed that the relaxation time $\tau _e$ is inversely proportional to $T_e$, and *G* parameter is independent of temperature. On the other hand, Hüttner^32^ suggested that $\tau _e$ carries an inverse relation to the amount of energy that is exchanged between electrons and the phonons. The theoratical concept was later derived by Kaganov et al.^33^ in which the Fermi-Dirac distribution and the Bose-Einstein distribution were assumed for electrons and phonons respectively. In their work, for electron temperatures on the order of 1 eV or greater, $\tau _e$ is no longer inversely proportional to the $T_e$ invalidating the use of the general form of Eq. (4) for the high electron temperature cases. Finally, the modified equation is expressed as^31^

5 $$\begin{aligned} G = G_{RT} \left[ \frac{A_e}{B_l}(T_e + T_l) + 1 \right] , \end{aligned}$$

where $A_e = (v_{ee} T_F^2)^{-1}$ and $B_l = (v_{ep} T_F)^{-1}$. $T_F$ is Fermi temperature, and $G_{RT}$ represents the *G* factor for room temperature. $v_{ee}$ and $v_{ep}$ denote collision rate of electron-electron (e-e) and electron-phonon (e-ph), respectively. The deduced *G* of Eq. (5) is linearly related to $T_e$, of which slope represents the collision ratio between e-e and e-ph. For bulk Au, *G* was analytically calculated to be 1.63 times greater than $G_{RT}$ at $T_e$ = 3500 K and $T_l$ = 300 K^31^, and was later experimentally measured as $G \approx 1.6G_{RT}$ when $T_e$ = 4000 K^34^. However, our result provides *G* = 84 $\times 10^{16}$ Wm$^{-3}$K$^{-1}$ that is approximately twice larger than the previously reported for a Cr single crystal^16,17^. We attribute this increase in the *G* value to the enhanced collision rate at higher temperature because higher energy electrons scatter more often according to kinetic theory^27,35^. However, we have not accounted for the additional complexity arising from the polycrystallinity of the film, since modified potentials at the surface and the grain boundaries are not available^36^.

### Film thermal conductivity

**Figure 4:**
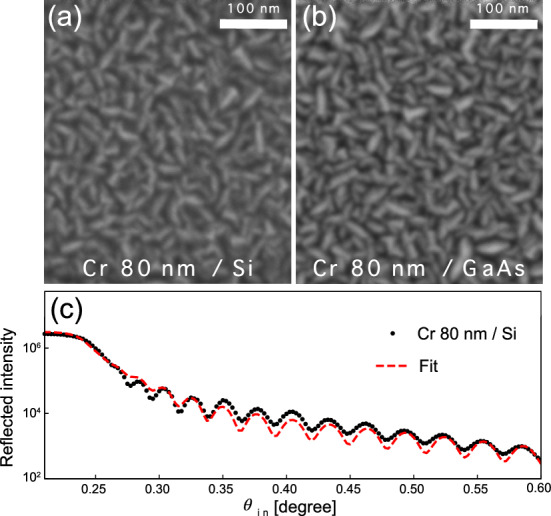
Scanning Electron Microscopy (SEM) images of 80 nm thickness of Cr film surfaces on the Si (**a**) and GaAs (**b**) as a substrate. (**c**) X-ray reflectivity measurement of Cr film on Si substrate with corresponding a model fitting. The interface roughness is estimated to be 10 Å.

Fitting our numerical model to data yields an effective electron thermal conductivity of $k_{e,eff}$ = 18.7 W m$^{-1}$K$^{-1}$, which is 5 times smaller than the value measured in a bulk system^18^. This discrepancy likely results from electron scattering at the grain boundaries in the polycrystalline film^37–39^. The effect of grain boundaries on energy relaxation and transport mechanisms in a polycrystalline metal film has been widely interpreted by the Matthiessen’s rule that describes the mean free path *l* of the system^40^:

6 $$\frac{1}{l} = \frac{1}{{l_{{crystal}} }} + \frac{1}{{l_{{grain}} }},$$

where $l_{crystal}$ and $l_{grain}$ are the mean free path lengths of the electrons in a single bulk crystal and polycrystalline grain. However, since multiple scattering events take place at the grain boundary, we employ the Mayadas and Shatzkes equation^41^ to calculate the ratio between the conductivity of a polycrystalline material, $k_{e,eff}$, and a single bulk crystal, $k_{e,crystal}$, with the probability *r* of reflection at a grain boundary.

7 $$\begin{aligned} \frac{k_{e,eff}}{k_{crystal}} = 1-\frac{3}{2} \beta + 3 \beta ^2 - 3\beta ^3 ln(1+\beta ^{-1}), \end{aligned}$$

where $\beta = \frac{l_{crystal}}{l_{grain}}(\frac{r}{1-r})$. Here, $k_{e,eff}$ is smaller than ${k_{crystal}}$ by approximately 20 $\%$ for *r* = 0.85, $l_{crystal}$ = 15 nm^42^, and $l_{grain}$ = 30 nm. The grain size of $l_{grain}$ is measured from a scanning electron microscopy image (see Fig. 4). This estimation for $k_e$ shows a good agreement with our experimental finding. We note that the film thickness can also contribute to lower the effective electron conductivity^43,44^. However, it is very challenging to separate the effects of interface and grain boundary scattering in thin polycrystalline films because the macroscopic scale assumptions are no longer valid at such microscopic length-scales.

### Substrate lattice thermal conductivity

As the size of the system becomes comparable to the mean free path (MFP) of phonons, the thermal conductivity can be significantly altered due to the size effects^45,46^. Such phenomenon has been widely reported for nanostructured materials such as nanowires or superlattices^47,48^. In addition, when the heat source and substrate are comprised of different materials, the thermal conductivity does not follow the Fourier theory^49^. In this case, the transport process can be better described by the quasi-ballistic transport model with Boltzmann transport equation^47,50,51^. Typically, the quasi-ballistic transport can be divided into two regimes, ballistic and diffusive transports. Transient surface reflectivity measurements based on pulsed near infrared laser have been widely used for such studies, where the measurement is primarily sensitive to dynamics on the metal surface due to the low photon energy^52–54^. Using X-ray diffraction, we made direct measurements of these two mechanisms. First, we have demonstrated the existence of the ballistic component, which manifests itself in the form of coherent interference patterns in reciprocal space, Brillouin scattering. This ballistic component is comprised of long-wavelength phonons and does not contribute to the heating of the lattice. Within the initial nanosecond, it propagates away from the metal-to-semiconductor interface. On the other hand, the effect of heat migration through diffusion emerges notably later. Table 1 shows our experimental results for the estimated thermal conductivity $k_s$ in the substrate and thermal boundary conductivity ($\sigma _{TBC}$) at the interface between the Cr film and the semiconductor substrates. We obtain approximately 4.3 and 11 times smaller conductivity values as compared to the bulk values for Si and GaAs, respectively. The deviations in the conductivity might be explained with a more elaborate heat transfer model between metal-nonmetal interface, taking into account the thermal properties of each phonon-branches^55^. Our result provides direct experimental evidence for the breakdown of Fourier’s Law. Ultimately, the correct calculation of the heat flux at heterostructure interfaces needs to account for the ballistically transported phonons as measured in this work. The overall picture of nanoscale thermal transport is incomplete without proper partitioning of energy between ballistic and diffusive phonons^7,50,52^.

## Conclusion

We investigate the laser-induced energy transport mechanism on a metal layered semiconductor system via high angular-resolution time-resolved X-ray diffraction. A careful transient diffraction peak analysis based on dynamical X-ray theory aided by the large dynamic range of our detection scheme enables modeling of the subtle features of scattering patterns over a wide *Q* range. By modeling this experimental data, we successfully measure the electron-phonon coupling constant *G* and thermal conductivity of electron $k_e$ of the metal layer despite the fact that typical lattice thermalization processes occur at much faster timescales than our temporal resolution available at a 3$^\text {rd}$ generation synchrotron radiation facility. We expect that additional measurements using the time-resolved X-ray diffraction technique combined with a more elaborate theoretical calculation will lead to improved microscopic descriptions of thermal transport in nanostructures, thereby opening new opportunities for improved material performance through the optimal control of energy carriers.

## Methods

Figure 1a shows a schematic of the experiment performed at the 7ID-C beamline at the Advanced Photon Source (APS)^56^ using 10 keV X-rays that were monochromatized by a water-cooled double-crystal diamond (111) monochromator. The X-rays were focused down to a spot size $d_\text {b}$ of 50 $\mu m^2$ by horizontal-plane focusing and a well-defined vertical slit. A near infrared laser system operating at a 5 kHz repetition rate was synchronized to the revolution frequency of the storage ring and delivered 50 fs optical pulses with a central wavelength of 800 nm. The relative temporal delay between X-ray and laser pulses was controlled with a timing jitter less than 2 ps, of which detail is elaborated elsewhere^57^. In our experimental geometry, the 4-circle diffractometer allowed us to measure the high-resolution rocking curve with a 0.5 millidegrees angular resolution. The 80-nm thick chromium metal layer deposited on the semiconductor substrates acted as a heat transducer. Transient changes in the symmetric (0 0 4) Bragg diffraction peaks of the substrate semiconductors (Si and GaAs) were acquired as a function of delay times between optical laser and X-ray pulses. The diffracted X-ray photons were collected by the customized Avalanche Photodiode (APD), which is capable of resolving the 153 ns electron bunch separation of APS in 24-bunch filling mode^58^.

### Sample preparation

The 80 nm of Cr films are deposited on the substrates by D.C. magnetron sputtering of a high purity chromium target in a chamber with a base pressure of about 7 $\times$ 10$^{-7}$ Torr. D.C. power for the sputtering was set to 57 W, and the substrates were not heated during deposition. Pure Ar gas was used as the working gas and maintained at 3.3 $\times$ 10$^{-3}$ Torr for sputtering the films. The morphology of the metal surface was investigated by SEM images shown in Fig. 4a and b, from which we estimate the grain size of approximately 30 nm. The thickness of the metal layer and interface roughness were estimated from X-ray reflectivity measurement to be 80 nm and 10 Å, respectively Fig. 4c.

### Calculation of a strain profile

In our model, the strain profile $\eta (z,t)$ in the substrate is comprised of two representative parts: (1) bipolar impulsive pulse which is mostly related to the mechanical properties of the system and (2) thermal diffusion which is governed by the thermal properties (see Fig. 3). The elastic wave equations for film and substrate can be expressed as^59^

8a $$\begin{aligned} \rho _{f,s}\frac{\partial ^2 u_{f,s}}{\partial ^2 t} = \frac{\partial \sigma _{f,s}}{\partial z}&= \rho _{f,s} v_{f,s}^2\frac{\partial ^2 u_{f,s}}{\partial z^2} - 3\alpha _{f,s}B_{f,s}\frac{\partial T_{f,s}}{\partial z}, \end{aligned}$$

8b $$\begin{aligned} \sigma _{f,s}&= \rho _{f,s}v_{f,s}^2\frac{\partial u_{f,s}}{\partial z}-3\alpha _{f,s}B_{f,s}\Delta T_{f,s}, \end{aligned}$$

where $u_{f,s}$, $\rho _{f,s}$, $\sigma _{f,s}$, $v_{f,s}$, $B_{f,s}$, and $T_{f,s}$ denote each film (*f*) and substrate (*s*) of the lattice displacement, mass density, total stress, speed of sound, bulk modulus, and lattice temperature, respectively. The second term in the right hand side of Eq. (8b) represents the stress source generated by thermalization process, turning out the impulsive pulses. In order to solve it the following boundary conditions were employed.

9a $$\begin{aligned} \sigma _f |_{z=0}&= 0, \end{aligned}$$

9b $$\begin{aligned} \sigma _f |_{z=d}&= \sigma _s |_{z=d},\end{aligned}$$

9c $$\begin{aligned} \frac{\partial u_s}{\partial z}\big |_{z = \text {end}}&= -\frac{1}{v_s}\frac{\partial u_s}{\partial t}\big |_{z = \text {end}},\end{aligned}$$

9d $$\begin{aligned} u_{f,s} = 0, ~&\frac{\partial u_{f,s}}{\partial t} = 0 ~ \text {at} ~ t = 0, \end{aligned}$$

where *d* is the film thickness. We assume zero stress at the film surface (Eq. 9a) due to the free end reflection at the surface, and continuous stress condition at the interface (Eq. 9b). In our time range, we only consider the impulsive waves propagating into the substrate and ignore the reflecting pulses from the end of the substrate (Eq. 9c). Equation (9d) defines the initial condition before the laser excitation. Subsequently, strain $\eta$ is evaluated as $\eta (z,t)=\partial u(z,t) {/} \partial z$. The lattice temperature distribution *T* is obtained by Eq. (2). The d’Alembert solution^60^ provides $f(z \pm vt)$ as the solution for the wave equation. In this calculation including TTM model, the finite difference method was employed to solve the governing equations (Eqs. 1, 2, 3, 8 and 9) with 2.67 nm of spatial resolution and 1.78 fs of temporal step.

### Dynamical X-ray diffraction simulation

In our modeling, the semiconductor substrates are treated as perfect single crystals. This is a reasonable assumption since the measured X-ray diffraction peaks are sufficiently narrow (13.68 and 18.36 arcsecond for Si and GaAs, respectively) despite the convolution effect from the instrumental broadening. The estimated mean crystalline domain sizes of 1.36 and 1.87 $\upmu$m are comparable to the X-ray extinction depth of the samples, e.g., 3.62 and 1.67 $\mu$m for Si and GaAs, respectively^61^. As a result, the multiple scattering of X-ray photons within the sample should be taken into account. We follow the numerical formulation that was initially derived by Bartels et al.^62^. in which X-ray diffractions from strained single crystals are calculated by solving the Takagi-Taupin equation. Owing to the large dynamic range available in our X-ray detection scheme, we are able to make a quantitative comparison between the calculated diffraction curve profile and data. We note that such in-depth shape profile analysis provides significant improvement in revealing fine details of atomic-scale strain distribution over the previous efforts, in which only Bragg peak centroid shifts were investigated^46,63–65^.

## Data Availability

All sample preparation conditions, characterization procedures, methods and data underlying in this paper are provided in the text. Any clarifications will be available by contacting the corresponding author on reasonable request.
